# Study of the Effect of Injection Bevacizumab through Various Routes in Neovascular Glaucoma

**DOI:** 10.5005/jp-journals-10008-1200

**Published:** 2016-08-05

**Authors:** Purvi R Bhagat, Kushal U Agrawal, Dipali Tandel

**Affiliations:** 1 Associate Professor, Department of Glaucoma Clinic, M&J Western Regional Institute of Ophthalmology, Ahmedabad, Gujarat, India; 2 Resident, Department of Glaucoma, M&J Western Regional Institute of Ophthalmology, Ahmedabad, Gujarat, India; 3 Student, Department of Glaucoma, M&J Western Regional Institute of Ophthalmology, Ahmedabad, Gujarat, India

**Keywords:** Anti-vascular endothelial growth factor, Bevacizumab, Glaucoma, Neovascular.

## Abstract

**Purpose:** To study the effect of injection bevacizumab on iris neovascularization (NVI) and angle neovascularization (NVA) and compare its efficacy in terms of visual outcome, NVI, NVA, and intraocular pressure (IOP) control between intracameral, intravitreal, and combined use.

**Materials and methods:** This was a prospective study conducted at a tertiary center for patients of neovascular glaucoma (NVG), including 20 eyes of 20 patients. After thorough evaluation, patients were divided into three groups: Intracameral, intravitreal, or combined, according to the route of injection bevacizumab required.

**Results:** About 30% of patients belonged to the age group 51 to 60 years of which 80% were female. In 50%, vein occlusion was the cause of NVG, and 50% needed intravitreal injection bevacizumab. After 4th week of injection 90% and after 12th week 60% were found to have absence of NVI. Patients who had IOP in the range of 11 to 20 mm Hg and 21 to 30 mm Hg showed lower IOP as compared to other groups. But no significant difference was noted in higher IOP groups. Only two patients required antiglaucoma surgery.

There was no statistically significant difference in visual outcomes in any groups. In all routes, there were statistically significant changes in NVI and NVG in the 1st and 4th weeks.

**Conclusion:** The effect of injection in all routes deteriorates after 8 weeks. Intracameral route of injection is found to be most effective in terms of control of IOP. There was no statistically significant difference in terms of improvement in best corrected visual acuity (BCVA) in any route. Injection bevacizumab is effective and statistically significant in reducing the need of antiglaucoma surgery for NVG patients.

**How to cite this article:** Bhagat PR, Agrawal KU, Tandel D. Study of the Effect of Injection Bevacizumab through Various Routes in Neovascular Glaucoma. J Curr Glaucoma Pract 2016;10(2):39-48.

## INTRODUCTION

Glaucoma is a group of diseases characterized by cupping and atrophy of optic nerve head and has attendant visual field loss related to increased or normal intraocular pressure (IOP). Glaucoma is the second leading cause of blindness worldwide.^1^

Glaucoma can be classified as

- Angle-closure glaucoma
- Open-angle glaucoma
- Developmental glaucoma.

Neovascular glaucoma (NVG) falls in the category of secondary angle-closure glaucoma. It is a serious disorder, which occurs as a late complication of ischemic and inflammatory retinopathies, tumors, and other causes.

First documented in 1871, historically it has been referred to as a hemorrhagic glaucoma, thrombotic glaucoma, congestive glaucoma, 100-day glaucoma, and diabetic hemorrhagic glaucoma.^2^ Central retinal vein occlusion (CRVO), proliferative diabetic retinopathy, carotid artery occlusive disease (CAOD), central retinal artery occlusion, retinoblastoma, malignant melanoma, and post retinal detachment surgery are major associated pathologies.

In Ischemic type of CRVO overall incidence of NVG is 40%.^3^ In diabetics, the incidence of neovascularization of iris (NVI) ranges from 1 to 17%.^4^ In proliferative diabetic retinopathy, the incidence of NVI goes up to 65% .^5^ Carotid artery occlusive disease is the third most common cause of NVG, accounting for at least 13% of cases.^6^

Hypoxia and poor retinal capillary circulation are believed to be the primary initiating events that lead to neovascularization and glaucoma. The evolution of NVG usually follows an ordered sequence beginning with new vessel formation and ending with fibrovascular membranes migrating over the drainage angle, potentially leading to open-angle and subsequent angle-closure and end-stage glaucoma.

Control of associated medical conditions (hypertension, diabetes mellitus), vascular endothelial growth factor (VEGF) inhibitors, panretinal photocoagulation, medical treatment for elevated IOP, conventional glaucoma filtering surgery with antifibrotics, glaucoma valve implant surgery, and cyclodestructive procedures are among the main treatment modalities.

It is now well known that VEGF plays a principal role in ocular conditions characterized by neovascu-larization.^7^ Activation of the VEGF-receptor pathway triggers signaling processes that promote growth of endothelial cells and their migration from preexisting vasculature.^8^

Bevacizumab (Avastin) is a humanized monoclonal antibody that binds to all isoforms of VEGF.^9^ Bevacizumab binds to receptor-binding domain of all VEGF-A isoforms. Consequently, it prevents the interaction between VEGF-A and its receptors (FLT-1 and KDR) on the surface of endothelial cells which starts the intercellular signaling pathway, leading to endothelial cell proliferation and new blood vessel formation. Regression of iris neovascularization after intravitreal injection of bevacizumab has been reported.^10,11^ This phenomenon has encouraged many surgeons to use VEGF inhibitors as a treatment for neovascular glaucoma.

There is high likelihood of profound vision loss once IOP rises and high incidence of major complications like anterior segment necrosis and phthisis bulbi. So, early diagnosis and treatment is key to preserve ocular function.

Although the effect of panretinal photocoagulation (PRP) is long lasting, it often takes several weeks to occur. During this period, progressive angle closure and optic nerve damage may ensue from elevated IOP, resulting in loss of vision.^12^

The aim of this study was to evaluate the effect of intravitreal, intracameral and combined (intravit-real + intracameral) bevacizumab injection in cases of neovascular glaucoma.

## MATERIALS AND METHODS

### Study Design

This was a prospective study conducted at a tertiary referral care centre for patients of NVG presenting between July 2012 and November 2014. It included 20 eyes of 20 patients of NVG.

### Inclusion Criteria

- Age group > 18 years
- Patients having visual potential at least perception of light
- Patients having NVI and or NVA
- Patients having IOP > 21 mm Hg.

### Exclusion Criteria

- Patients previously given PRP or intravitreal triam-cinolone or anterior retinal cryopexy
- Patients having corneal edema not permitting visualization of angles by gonioscopy.

All patients included in our study underwent the following set of examinations:

- Detailed history.
- Examination of anterior segment with torchlight and slit lamp.
- Tonometry using Perkins’s handheld applanation tonometer.
- Gonioscopy using Goldman two-mirror goniolens and grading as per Shaffer’s classification.
- Detailed fundus examination using direct and indirect ophthalmoscope and slit lamp biomicroscopy by + 78D and + 90D lens to assess disk and associated retinal pathology.
- Optical coherence tomography (OCT), macular scan, and optic nerve head (ONH) scan, in patients clinically having macular edema, using the Topcon 2000 SD-OCT system which acquires 26,000 axial scans (A-scans) per second and has a 6-μm depth resolution (full-width half-maximum) in tissue. The scan pattern consists of 128 × 512 pixels taken in 7 × 7 mm^2^ area with an interval of 0.05 mm.
- Fundus fluorescein angiography (FFA) in cases of ischemic NVG by Zeiss Visulas machine.

After evaluation, patients were divided into three groups: Intracameral, intravitreal or intracameral + intravitreal (combined) according to route of injection bevacizumab required. Informed consent was taken from all patients before all interventions.

### Method of Intravitreal Injection Bevacizumab

- Topical proparacaine 0.5% instilled in eyes three times 5 minutes apart prior to injection
- Eyes painted with solution of 5% povidone iodine and draped
- Injection bevacizumab 1.25 mg (0.05 mL) given 4, 3.5, or 3 mm away from limbus in phakic, pseudophakic, and aphakic eye respectively in inferior temporal quadrant through 26 no. needle
- Surface wash with gentamicin given.

### Method of Intracameral Injection Bevacizumab

- Preparation of patients was same as in intravitreal group.
- Following this, patients were given 1.25 mg (0.05 mL) in anterior chamber through 26 no. needle.

### Method of Combined Route of Injection Bevacizumab

- Preparation of patients was same as above.
- After that patients were given 1.25 mg (0.05 mL) intravitreal bevacizumab followed by 1.25 mg (0.05 mL) intracameral bevacizumab via 26 no. needle and same method as above.

Among 20 patients, 8 patients were given intracameral bevacizumab, 10 were given intravitreal bevacizumab, and 2 were given combined intravitreal + intracameral. Patients were followed on the next day and at 1st, 2nd, 4th, 8th, and 12th weeks.

After Injection, according to IOP, patients were given antiglaucoma medications like topical beta-blocker, alpha-agonist, and carbonic anhydrase inhibitors and oral acetazolamide as per requirement. In patients with inflammation due to NVI and having corneal edema, topical difluprednate eye drops four times a day and cycloplegic like atropine eye ointment three times a day were given.

In patients with ischemic NVG, photocoagulation of retina by argon green laser was performed. In two patients with uncontrolled IOP despite injection beva-cizumab and antiglaucoma medication, conventional trabeculectomy with mitomycin C was performed. In two patients, peripheral iridotomy, using Nd-YAG laser, was performed. In two patients with uncontrolled IOP and no useful vision, cyclocryotherapy was performed. In one patient, 25G pars-plana vitrectomy using Constellation machine and endolaser PRP using endolaser probe of same machine was performed.

Data were analyzed in terms of regression of neovascularization, IOP control, visual outcomes by Student’s t-test for qualitative data, and Mcnemar’s test for quantitative data with significance of test kept at p-value < 0.05. Prognosis was noted in terms of requirement of further surgical or medical management.

## RESULTS

**Graph 1:**
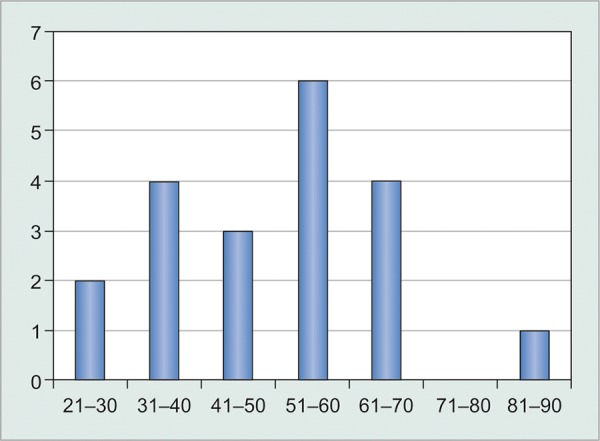
Number of subjects in various age groups

- Agewise distribution (Table 1 and Graph 1)

**Table 1:** **Table 1:** Number of subjects in each age group

*Age in years*		*Number of patients*		*Percentage*	
21-30		2		10	
31-40		4		20	
41-50		3		15	
51-60		6		30	
61-70		4		20	
71-80		0		00	
81-90		1		05	

Twenty subjects in the age group from 21 to 90 were studied. Maximum subjects (30%) belonged to the age group 51 to 60 years.

**Graph 2:**
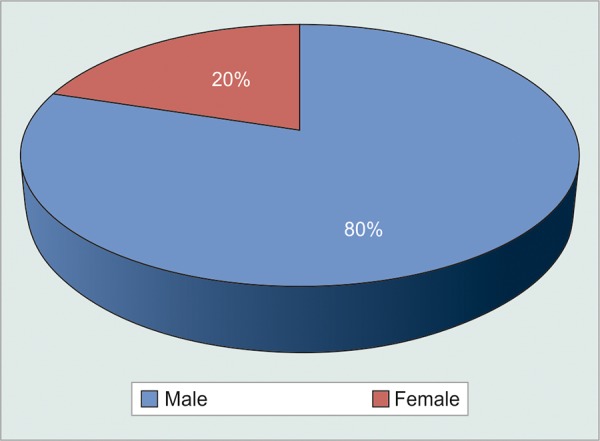
Gender distribution

**Graph 3:**
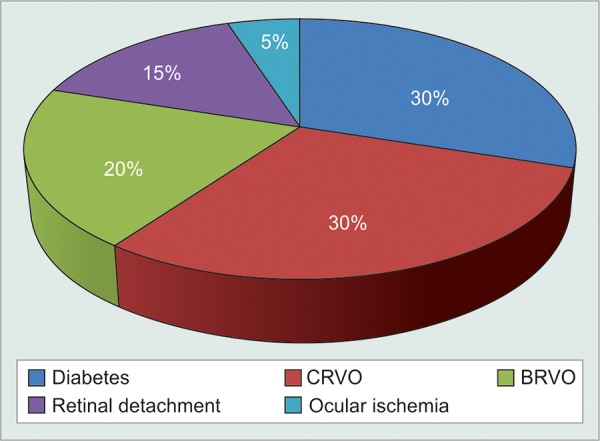
Distribution of diseases

- Gender distribution (Table 2 and Graph 2)
- Distribution of diseases causing NVG (Table 3 and Graph 3)

**Table 2:** **Table 2:** Number of male and female subjects

*Sex*		*Number*		*Percentage*	
Male		16		80	
Female		04		20	

Of 20 patients, vein occlusion as a cause of NVG, present in 10 (50%) patients, was found to be the most common association, followed by diabetes in 6 (30%) of patients, retinal detachment in 3 (15%) of patients, and ocular ischemia in 1 (5%) patient.

**Graph 4:**
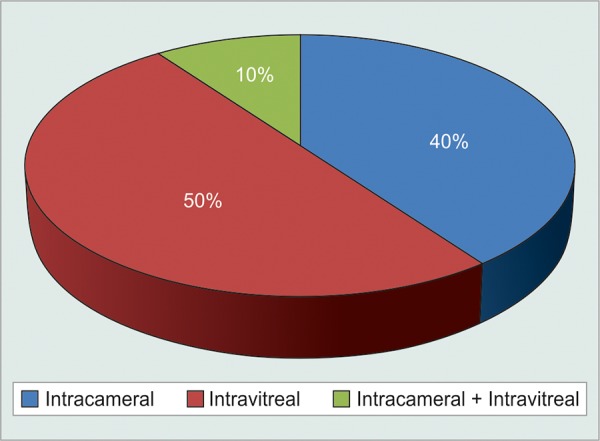
Routes of injection

- Routes of injection bevacizumab (Table 4 and Graph 4)

Thus majority of patients 10 (50%) needed intravitreal injection bevacizumab.

**Graph 5:**
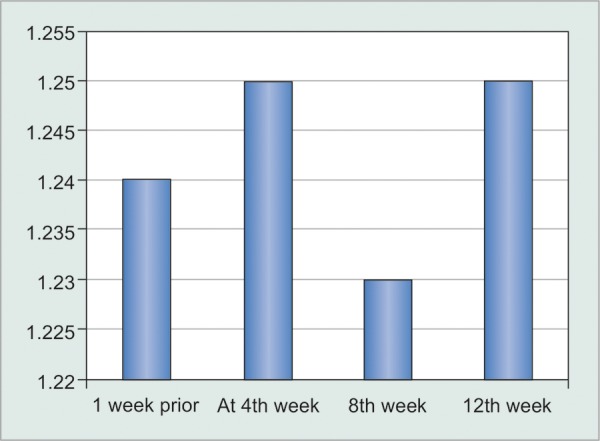
Visual acuity changes in BCVAlogMAR

- Visual outcomes after injection bevacizumab (Table 5 and Graph 5)

The mean preoperative BCVA logarithm of minimal angle of resolution (BCVA logMAR) was 1.24. At the end of 4th, 8th, and 12th weeks, it was around 1.25, 1.23 and 1.23 respectively.

**Table 3:** **Table 3:** Distribution of diseases

*Diseases*		*Number of* * patients*		*Percentage*	
Diabetes		6		30	
Central retinal vein occlusion		6		30	
Branch retinal vein occlusion		4		20	
Retinal detachment		3		15	
Ocular ischemia		1		05	

**Table 4:** **Table 4:** Routes of injection of Bevacizumab

*Route of injection*		*Number of* * patients*		*Percentage of* * patients*	
Intracameral		08		40	
Intravitreal		10		50	
Intracameral + intravitreal		02		10	

**Table 5:** **Table 5:** Visual acuity changes in BCVAlogMAR

*Time period*		*BCVA logMAR*		*p-value*		*Significance*	
1st week prior		1.24		–		–	
At 4th week		1.25		0.48		NS	
8th week		1.23		0.48		NS	
12th week		1.25		0.48		NS	

NS: Not significant

In one patient there was significant drop of vision from 6/36 (logMAR 0.8) to CF 3 mt (logMAR 1.3), which was due to re-detachment of retina operated for retinal detachment. In another patient BCVA dropped from 6/60 (logMAR 1.0) to CF 2 mt (logMAR 1.4), which was due to re occurrence of NVI, and associated corneal edema. In rest of the patients there was no statistically significant difference noted in terms of visual acuity.

Thus, assessment of vision showed that there is no statistically significant change in the vision at the end of follow-up.

- Effect on neovascularization of iris

The p value calculated by Mcnemar’s test, in comparison with presence of NVI before 1 week of injection bevaci-zumab (Table 6).

**Graph 6:**
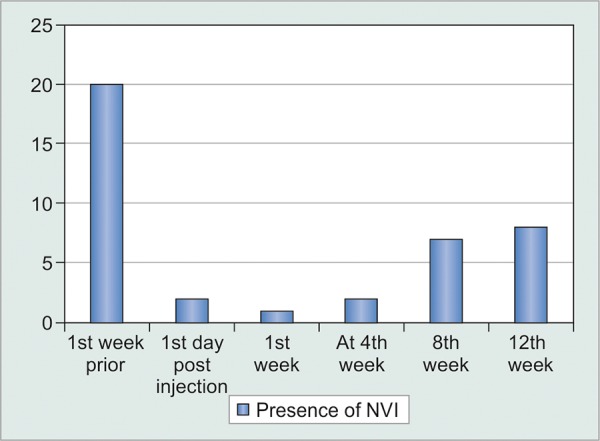
Number of subjects having iris neovascularization

**Graph 7:**
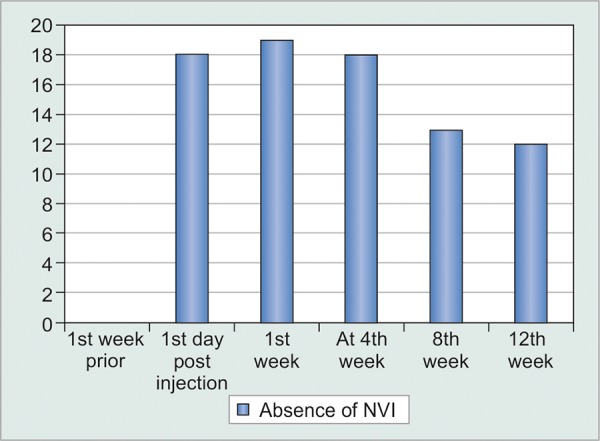
Number of subjects showing absence of iris neovascularization after Injection Bevacizumab

So in terms of effect of injection bevacizumab on NVI; there was statistically significant difference noted in control of NVI post injection up to 12 weeks (Graphs 6 and 7).

So, after injection from day 1, 1st and 4th weeks, there were 90, 95, and 90% cases found to have absence of NVI. In comparison at 8th and 12th weeks 65 and 60% cases were found to have absence of NVI.

**Graph 8:**
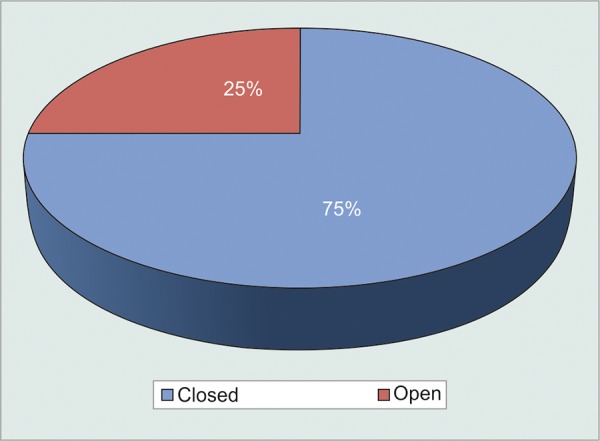
Status of angles on gonioscopy

- Gonioscopy grading (Graph 8)

Out of 20 patients, 15 (75%) were having closed angle and 5 (25%) were having open angle.

**Table 6:** **Table 6:** Number of subjects having iris neovascularization

*Duration*		*Presence of neovascularization*		*Percentage of presence*		*p-value*		*Clinical significance*	
1 week prior		20		100		–		–	
1st day post injection		2		10		0.000022		S	
1st week		1		5		0.000013		S	
4th week		2		10		0.000022		S	
8th week		7		35		0.000311		S	
12th week		8		40		0.0005		S	

S: Significant

**Graph 9:**
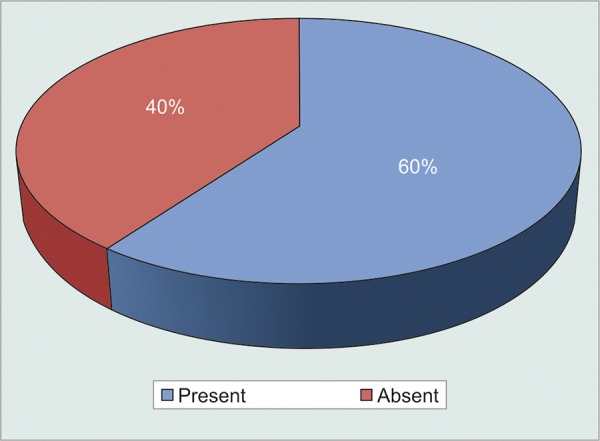
Number of subjects with angle closure showing neovascularization

Among 15 angle-closure patients, neovascularization was visible in 9 (60%) and not visible in 6 (40%) patients. But, in open-angle NVG patients, NVA was not visible in any of the patients (Graph 9).

After injection bevacizumab, none of the patients having angle closure reverted to open-angle stage. • Effect of injection bevacizumab on NVA (Table 7)

At 1st-, 4th-, and 8th-week post injection, there was statistically significant difference noted in comparison to 1 week prior result; but not at 12th week. So, injection bevacizumab was found to be effective up to 8th week in cases of NVA; but not up to 12th week.

**Table 7:** **Table 7:** Effect of Injection Bevacizumab on NVA

*Duration*		*Presence* * of NVA*		*Absence* * of NVA*		*p-value*		*Significance*	
1 week prior		9		0					
At 1st week		0		9		0.0027		S	
4th week		0		9		0.0027		S	
8th week		3		6		0.01		S	
12th week		6		3		0.08		NS	

S: Significant; NS: Not significant

- Effect of Injection bevacizumab on intraocular pressure (Table 8)

Thus, in comparison to number. of patients 1 week prior to Injection Bevacizumab in the range of 11 to 20 and 21 to 30 mm Hg to post injection at 1st and 4th week, there was statistically significant difference noted in terms of shift of patient to lower IOP group.

But, in IOP group 31 to 40 mm Hg and 41 to 50 mm Hg there was no statistically significant difference noted in terms of shift of patient to lower IOP group (Table 9).

Thus, clinically significant difference can be noted in group 11 to 20 mm Hg and 21 to 30 mm Hg, but not in higher IOP range groups.

- Final Outcome
- Among 7 patients who developed NVI again:

Three patients were given repeat Injection Bevacizumab, out of these three patients, two were given laser PRP and maintained on antiglaucoma medication; and one patient despite repeat injection bevacizumab, laser PRP and anti-glaucoma Medication did not show control of IOP and had to undergo antiglaucoma surgery with mitomycin C and maintained IOP control post surgery.

Among the other four patients with reappearance of NVI, three underwent laser PRP and maintained IOP with antiglaucoma Medication and one patient with retinal detachment had to undergo vitrectomy with endolaser PRP and maintained on antiglaucoma medications.

- Among rest 13 patients who did not develop reappearance of NVI:

Two patients had to undergo antiglaucoma surgery with mitomycin C, two patients were treated with laser peripheral iridotomy with laser PRP and two patients underwent Cyclocryotherapy for IOP control (Table 10).

Thus, there was statistically significant difference noted in terms of need of surgical *vs* medical control of IOP after injection bevacizumab.

**Graph 10:**
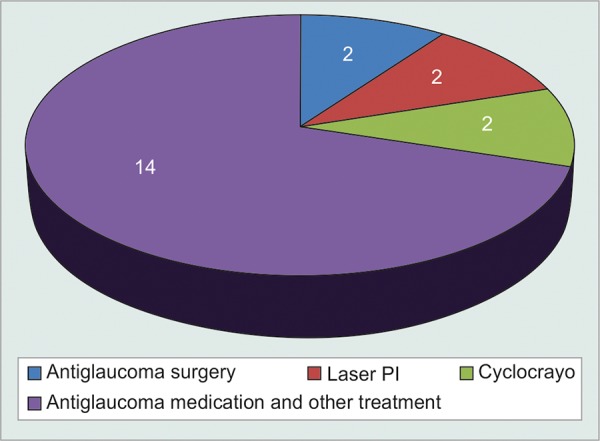
Final outcome of 20 patients

Among rest eight patients, six were maintained on antiglaucoma medication and laser PRP. Among two patients with retinal detachment, one patient having chronic retinal detachment with vision PL + PR defective was maintained on antiglaucoma medication alone and other one eyed patient with retinal detachment not involving macula having BCVA 6/36 was kept under follow up with IOP maintained on antiglaucoma medications (Graph 10).

Out of 14 patients maintained on antiglaucoma medications, 6 were on single drug and 10 were on two groups of antiglaucoma medications.

**Table 8:** **Table 8:** Comparison of number of patients shifting to lower IOP group and their significance

*Range of IOP*		*Number of patients* *1 week prior*		*At 1st week*		*p-value*		*Significance*		*At 4th week*		*p-value*		*Significance*	
11-20		00		09		0.002		S		08		0.004		S	
21-30		16		09		0.008		S		09		0.008		S	
31-40		04		01		0.08		NS		02		0.16		NS	
41-50		00		01		0.31		NS		01		0.31		NS	

S: Significant; NS: Not significant

**Table 9:** **Table 9:** Changes in IOP

*Range of IOP*		*Number of patients* *1 week prior*		*At 8th week*		*p-value*		*Significance*		*At 12th week*		*p-value*		*Significance*	
11-20		00		07		0.008		S		08		0.004		S	
21-30		16		10		0.014		S		09		0.008		S	
31-40		04		2		0.16		NS		2		0.16		NS	
41-50		00		01		0.31		NS		01		0.31		NS	

S: Significant; NS: Not significant

**Table 10:** **Table 10:** Final treatment modalities

*Total* * number* * of* * patients*		*Maintained* * IOP control on* * laser PI and* * antiglaucoma* * medication*		*Required* * surgery or* * cyclocryotherapy*		*p-value*		*Significance*	
20		16		04		0.04		S	

S: Significant

### Comparison between Efficacy of Intracameral, Intravitreal and Intracameral + Intravitreal (Combined) Routes of Injection

- Visual outcomes (Table 11A)

**Table 11A:** **Table 11A:** Assessment of vision in between three routes

*Route of injection*		*1 week prior mean* * logMAR vision*		*At 12th week mean* * logMAR vision*		*p-value comparison between* * 1 week prior and at 12th week*		*Significance*	
Intracameral		1.23		1.31		0.26		NS	
Intravitreal		1.20		1.16		0.4		NS	
Combined		1.45		1.45		0.5		NS	

NS: Not significant

**Table 11B:** **Table 11B:** Change in NVI on follow-up

*Route*		*Patients of NVI* * 1 week prior*		*At 4th week* * presence*		*p-value*		*Significance*		*At 4th week* * presence*		*p-value*		*Significance*	
Intracameral		8		0		0.004		S		1		0.008		S	
Intravitreal		10		1		0.003		S		1		0.003		S	
Combined		2		00		0.002		S		0		0.002		S	

S: Significant

There was no statistically significant difference in visual outcomes in all three groups at the end of study.

- Iris Neovascularization (Table 11B)

Thus, in all routes there was statistically significant change in NVI post injection bevacizumab noted at 1st and 4th week. But, in Combined group at 8th and 12th week there was no statistically significant change noted.

- Angle Neovascularization (Table 11C)

Thus, in terms of control of NVA post injection bevacizumab, there was statistically significant difference noted in all routes at 1st and 4th week, but not at 8th week or 12th week. At 8th week significance was noted only in Combined group.

- Intraocular Pressure
- Intracameral group (Table 12A)

Thus, in Intracameral group there was statistically significant difference noted in terms of shift of patients to lower IOP group at 4th, 8th and 12th week. But, not at 1st week in 11 to 20 mm Hg and 21 to 30 mm Hg IOP group. No statistically significant difference was noted in 31 to 40 mm Hg and 41 to 50 mm Hg IOP group.

- Intravitreal group (Table 12B)

**Table 11C:** **Table 11C:** Change in NVA in total number of patients on follow-up

*Route*		*1 week prior*		*At 1st week* *presence*		*p-value*		*Significance*		*At 4th week* *presence*		*p-value*		*Significance*	
Intracameral		4		0		0.04		S		0		0.04		S	
Intravitreal		3		0		0.03		S		0		0.03		S	
Combined		2		0		0.04		S		0		0.04		S	
S: Significant															
*Route*		*1 week prior*		*At 8th week* *presence*		*p-value*		*Significance*		*At 12th week* *presence*		*p-value*		*Significance*	
Intracameral		4		2		0.16		NS		4		–		NS	
Intravitreal		3		1		0.08		NS		2		0.15		NS	
Combined		2		0		0.04		S		1		0.31		NS	

NS: Not significant; S: Significant

**Table 12A:** **Table 12A:** Comparison of number of patients shifting to lower IOP group and their significance

*Range of IOP*		*Number of patients* *1 week prior*		*At 1st week*		*p-value*		*Significance*		*At 4th week*		*p-value*		*Significance*	
11-20		0		3		0.08		NS		5		0.02		S	
21-30		7		4		0.08		NS		2		0.02		S	
31-40		1		1		0.31		NS		1		0.31		NS	
41-50		0		0		–		NA		0		–		NA	
	
*Range of IOP*		*Number of patients* *1 week prior*		*At 8th week*		*p-value*		*Significance*		*At 12th week*		*p-value*		*Significance*	
11-20		0		4		0.04		S		5		0.02		S	
21-30		7		3		0.04		S		2		0.02		S	
31-40		1		1		0.31		NS		1		0.31		NS	
41-50		0		0		–		NA		0		–		NA	

NS: Not significant; NA: Not applicable; S: Significant

**Table 12B:** **Table 12B:** Comparison of number of patients shifting to lower IOP group and their significance

*Range of IOP*		*Number of patients* *1 week prior*		*At 1st week*		*p-value*		*Significance*		*At 4th week*		*p-value*		*Significance*	
11-20		0		3		0.08		NS		2		0.15		NS	
21-30		7		6		0.31		NS		6		0.31		NS	
31-40		3		1		0.15		NS		2		0.31		NS	
41-50		0		0		–		NA		0		–		NA	
	
*Range of IOP*		*Number of patients* *1 week prior*		*At 8th week*		*p-value*		*Significance*		*At 12th week*		*p-value*		*Significance*	
11-20		0		2		0.15		NS		2		0.15		NS	
21-30		7		6		0.31		NS		6		0.31		NS	
31-40		3		2		0.31		NS		2		0.31		NS	
41-50		0		0		–		NA		0		–		NA	

NS: Not significant; NA: Not applicable

**Table 12C:** **Table 12C:** Comparison of number of patients shifting to lower IOP group and their significance

*Range of IOP*		*Number of patients* *1 week prior*		*At 1st week*		*p-value*		*Significance*		*At 4th week*		*p-value*		*Significance*	
11-20		0		1		0.31		NS		1		0.31		NS	
21-30		2		1		0.31		NS		1		0.31		NS	
31-40		0		0		–		NS		0		–		NS	
41-50		0		0		–		NA		0		–		NA	
	
*Range of IOP*		*Number of patients* *1 week prior*		*At 8th week*		*p-value*		*Significance*		*At 12th week*		*p-value*		*Significance*	
11-20		0		1		0.31		NS		1		0.31		NS	
21-30		2		1		0.31		NS		1		0.31		NS	
31-40		0		0		–		NS		0		–		NS	
41-50		0		0		–		NA		0		–		NA	

NS: Not significant; NA: Not applicable

Thus, in Intravitreal group there was no statistically significant difference noted in terms of IOP lowering in any IOP group at any duration.

- Intracameral + intravitreal group (Table 12C)

Thus, in intracameral + intravitreal group also, there was no statistically significant difference noted in terms of IOP lowering in any IOP group at any duration.

## DISCUSSION

In our study, vein occlusion was the most common association found as a cause of NVG; 50% of patients had vein occlusion as a primary cause of NVG. It also correlates with the study done by Hayreh et al^3^ who also noted 40% of cases of vein occlusion being associated with neovascularization.

In our study, at the end of 12th week there was no statistically significant difference noted in BCVA as compared to 1 week prior in all routes of injection beva-cizumab. In only two patients (10%), there was worsening of vision, in one patient due to repeat retinal detachment after vitrectomy and in one branch retinal vein occlusion (BRVO) patient due to worsening of visual hallucinations (VH) on follow-up.

In a study done by Asaad A. Ghanem et al,^11^ at the end of follow-up, the visual acuity improved in nine (56.25%) cases, worsened in one (6.25%) case, and remained at the same level in six (37.5%) cases. Improvement was mainly due to clearing of ocular media. Vitreous hemorrhage improved in four cases, and hyphema disappeared in one case. Only one case with significant vitreous hemorrhage (case no. 2) resisted clearance within the follow-up period.

In a study by Abeer Khattab et al,^13^ BCVA showed noticeable improvement during the course of follow-up; the mean preoperative BCVA logarithm of minimal angle of resolution (BCVA logMAR) was 1.47 ± 0.511, which significantly improved to 1.29 ± 0.55, 1.16 ± 0.58, 1.15 ± 0.6, and 1.3 ± 0.56 at 1 week and 1, 2, and 3 months, postoperatively respectively.

But, in latter study, laser PRP was also mainstay of therapy along with injection bevacizumab, and in a former study, out of 16 patients 8 were given laser PRP before injection bevacizumab. As laser PRP is a confounding factor in clinical assessment of IOP, BCVA, and NVI, it was not given as a primary mode of therapy in our study, up to 12 weeks of follow-up; except in one case of PDR, in which despite injection bevacizumab and antiglaucoma medications, there was recurrence of NVI and uncontrolled IOP with useful vision. All other patients were maintained on antiglaucoma medications and definitive management like laser PRP, antiglaucoma surgery, laser peripheral iridotomy, cyclocryotherapy were done at the end of follow-up.

In a former study, media clarity was the most common reason associated with increased BCVA. But, in our study, only one patient having BRVO with vitreous hemorrhage, worsened visually on follow-up due to recurrence of NVI and VH. Corneal edema as an associated factor of decreased vision was present in three patients in whom vision was defective projection of rays and which did not improve post injection. In all other patients, media was clear.

In our study, statistically significant regression of NVI post injection was noted up to 12 weeks of study, and statistically significant regression of NVA was noted up to 8 weeks. At the end of 4th week, regression of NVI was noted in up to 90% of patient in our study, but regression was in 65 and 60% respectively at 8th and 12th weeks.

In our study, all routes of injection bevacizumab showed statistically significant regression of NVI and NVA up to 4 weeks and showed variable results thereafter.

Our study also correlates with a study done by Ghanem et al^11^ wherein at the 2nd follow-up day, there was complete regression of NVI in 100% of cases. Complete regression was noted in 37.5% of eyes at 8th week. Thus, in terms of regression on NVI, as in our study, this study also showed reappearance of NVI at 8th week in majority of cases.

In our study, there was statistically significant difference in terms of number of patients shifting to lower IOP group and mean IOP at the end of 12th week; in 21 to 30 mm Hg group but not in 31 to 40 mm Hg group.

In a study by Khattab et al,^13^ in which bevacizumab was given by combined route followed by laser PRP; the preoperative mean IOP was 58.7 ± 16.2 mm Hg; it decreased to 20.8 ± 9.99 mm Hg at 1 week, 18.47 ± 5.6 mm Hg at 1 month, 22.65 ± 8.7 mm Hg at 2 months, and 25.34 ± 6.806 mm Hg at 3 months postoperatively.

Higher IOP associated with synechiae of angle structures, which it cannot be reverted by injection bevaci-zumab, is the most probable reason of this. Besides this, the above study did not show results of IOP control in terms of range of IOP, and it showed mean of IOP of all patients and laser PRP was given before completion of study in patients which can be a confounding factor in that study.

To compare intravitreal, intracameral, and combined group, in our study in terms of control of IOP, intracameral route was found to be more effective in 21 to 30 mm Hg IOP range. In our study also, out of 20 only 4 patients required antiglaucoma surgery or cyclocryotherapy.

A study by Chalam et al^14^ also found that out of nine patients, eight were having control of IOP with antiglau-coma medications without need of antiglaucoma surgery after intracameral injection bevacizumab.

## CONCLUSION

- There was no statistically significant difference noted in terms of improvement in BCVA in any routes of injection bevacizumab.
- Effect of injection bevacizumab in terms of regression of NVI is marked up to 4 to 8 weeks and then starts deteriorating in all routes of injection.
- In intracameral route of injection, statistically significant difference in terms of control of IOP has been noted up to 12 weeks in IOP range 21 to 30 mm Hg, but not in 31 to 40 mm Hg. In intravitreal and combined group difference in terms of control of IOP was not statistically significant at 4th, 8th and 12th weeks. So, intracameral route was found to be most effective in terms of control of IOP, and there was no advantage of combined route over it noted.
- Of 20 patients, at the end of follow-up, only 2 patients required antiglaucoma surgery and 2 patients required cyclocryotherapy, rest 16 patients were maintained IOP with antiglaucoma medications, and with 2 patients requiring laser peripheral iridotomy.

So, injection bevacizumab has been found to be effective and statistically significant to decrease requirement of NVG patients for antiglaucoma surgery.

Though the sample size is small, it definitely yields substantial results which can help in managing a blinding disease like NVG in a better way.
